# A Negative Allosteric Modulator for α5 Subunit-Containing GABA Receptors Exerts a Rapid and Persistent Antidepressant-Like Action without the Side Effects of the NMDA Receptor Antagonist Ketamine in Mice

**DOI:** 10.1523/ENEURO.0285-16.2017

**Published:** 2017-03-07

**Authors:** Panos Zanos, Mackenzie E. Nelson, Jaclyn N. Highland, Samuel R. Krimmel, Polymnia Georgiou, Todd D. Gould, Scott M. Thompson

**Affiliations:** 1 Department of Psychiatry, University of Maryland School of Medicine, Baltimore, MD 21201; 2 Department of Physiology, University of Maryland School of Medicine, Baltimore, MD 21201; 3 Department of Pharmacology, University of Maryland School of Medicine, Baltimore, MD 21201; 4 Department of Anatomy and Neurobiology, University of Maryland School of Medicine, Baltimore, MD 21201; 5 Program in Neuroscience, University of Maryland School of Medicine, Baltimore, MD 21201; 6 Program in Toxicology, University of Maryland School of Medicine, Baltimore, MD 21201

**Keywords:** antidepressant, behavior, depression, GABA-NAM, γ oscillation, ketamine

## Abstract

New antidepressant pharmacotherapies that provide rapid relief of depressive symptoms are needed. The NMDA receptor antagonist ketamine exerts rapid antidepressant actions in depressed patients but also side effects that complicate its clinical utility. Ketamine promotes excitatory synaptic strength, likely by producing high**-**frequency correlated activity in mood-relevant regions of the forebrain. Negative allosteric modulators of GABA-A receptors containing α5 subunits (α5 GABA-NAMs) should also promote high**-**frequency correlated electroencephalogram **(**EEG) activity and should therefore exert rapid antidepressant responses. Because α5 subunits display a restricted expression in the forebrain, we predicted that α5 GABA-NAMs would produce activation of principle neurons but exert fewer side effects than ketamine. We tested this hypothesis in male mice and observed that the α5 GABA-NAM MRK-016 exerted an antidepressant-like response in the forced swim test at 1 and 24 h after administration and an anti-anhedonic response after chronic stress in the female urine sniffing test (FUST). Like ketamine, MRK-016 produced a transient increase in EEG γ power, and both the increase in γ power and its antidepressant effects in the forced swim test were blocked by prior administration of the AMPA-type glutamate receptor antagonist 2,3-dioxo-6-nitro-1,2,3,4-tetrahydrobenzo[*f*]quinoxaline-7-sulfonamide (NBQX). Unlike ketamine, however, MRK-016 produced no impairment of rota-rod performance, no reduction of prepulse inhibition (PPI), no conditioned**-**place preference (CPP), and no change in locomotion. α5 GABA-NAMs, thus reproduce the rapid antidepressant-like actions of ketamine, perhaps via an AMPA receptor (AMPAR)-dependent increase in coherent neuronal activity, but display fewer potential negative side effects. These compounds thus demonstrate promise as clinically useful fast-acting antidepressants.

## Significance Statement

There is need for new, fast-acting antidepressant drugs. The NMDA receptor antagonist ketamine exerts rapid antidepressant actions but has many side effects. We predicted that drugs that weaken inhibitory synaptic transmission by modulating GABA_A_ receptors containing α5 subunits would exert rapid antidepressant effects but cause fewer side effects because they act at one type of receptor with a restricted brain localization. Indeed, administration of an α5 GABA-NAM resulted in rapid and persistent antidepressant effects in several behavioral tests and did not affect locomotion or motor performance, did not change a measure of psychosis susceptibility, and did not appear to have abuse potential. These compounds thus display great clinical promise as novel fast-acting antidepressants.

## Introduction

Selective serotonin reuptake inhibitors (SSRIs), the current standard medicinal treatment for major depression, are effective in only some patients and typically require many weeks to produce their therapeutic effects (Rush et al., 2006; Gaynes et al., 2009). The discovery that the NMDA**-**type glutamate receptor (NMDAR) antagonist ketamine reverses symptoms in patients with treatment-resistant depression within hours of administration and that these effects persist for many days (Berman et al., 2000; Zarate et al., 2006; Singh et al., 2016) suggests that faster acting alternatives to SSRIs can be identified. Unfortunately, acute and chronic ketamine use can elicit many undesirable effects in humans, including acute psychosis, psychomotor activation, loss of consciousness, cognitive impairment, dissociation, and abuse potential, rendering it poorly suited for wide-spread use in the treatment of depression (Morgan et al., 2004; Pomarol-Clotet et al., 2006; Wolff and Winstock, 2006; Morgan et al. 2012). A better understanding of the mechanisms by which ketamine exerts its antidepressant actions is underway to develop other novel antidepressants that can act rapidly to produce a persistent relief of depressive symptoms without the side effect profile of ketamine.

There is considerable evidence that ketamine and its active metabolite, hydroxynorketamine, produce their antidepressant actions by strengthening excitatory glutamatergic synaptic transmission involving AMPA receptors (AMPARs) (Maeng et al., 2008; Li et al., 2010; Autry et al., 2011; Koike et al., 2011; 2014; Zanos et al., 2016) and that defects in excitatory transmission in brain regions controlling reward and mood contribute to the pathology of depression (Pittenger and Duman, 2008; Lim et al., 2012; Yuen et al., 2012; Kallarackal et al., 2013; Thompson et al., 2015). A key mechanism underlying the rapid antidepressant actions of ketamine in rodents (e.g., Kittelberger et al., 2012; Middleton et al., 2008; Hunt et al., 2011) and in humans (Cornwell et al., 2012; Driesen et al., 2013), as well as its hydroxynorketamine metabolites [e.g., (2R,6R)-HNK; Zanos et al., 2016], may be a transient period of high**-**frequency cortical activity, characterized by an increased power in the γ frequency band as measured by electroencephalogram (EEG).

Weakening the strength of GABAergic inhibitory synaptic transmission is another means to increase excitatory interactions between principle cells and might therefore exert a ketamine-like antidepressant response. Negative allosteric modulators of GABA type A receptors (GABA_A_Rs) directed selectively against receptors containing α5 subunits (α5 GABA-NAMs), including MRK-016 and L-655 781, exert a rapid antianhedonic behavioral response and restore the strength of stress-sensitive hippocampal synapses in several rat chronic stress models (Fischell et al., 2015). Whether α5 GABA-NAMs exert traditional antidepressant actions and whether they promote a ketamine-like increase in EEG γ activity is not known. In addition, investigation of the side effect profile of α5 GABA-NAMs in rodents has not yet been published. Unlike the widespread distribution of NMDA receptors targeted by ketamine, which may account for its side effects, GABA_A_Rs containing α5 subunits have a very limited distribution in the brain (Malherbe et al., 1990; Persohn et al., 1992). It can therefore be predicted that α5 subunit**-**selective α5 GABA-NAMs will exert comparatively fewer adverse side effects.

We therefore tested the hypotheses that acute administration of α5 GABA-NAMs would produce rapid and persistent antidepressant effects via AMPAR-mediated increases in EEG γ power similar to those evoked by ketamine but that it would not produce psychotomimetic and reward behaviors like those induced by ketamine, due to lack of NMDAR inhibition.

## Materials and Methods

### Animals

Male CD-1 mice (seven weeks old on arrival, Charles River Laboratories) were housed in groups of four to five per cage (unless otherwise stated) with a 12/12 h light/dark cycle (lights on at 07:00 A.M.), in a temperature- and humidity-controlled environment. Food and water were available *ad libitum*. Mice acclimatized to the new environment for 7 d before the start of the experiments. For the chronic restraint stress (CRS) and conditioned-place preference (CPP) experiments, male C57BL/6J mice (eight weeks old) were used. For obtaining the urine for the female urine sniffing test (FUST), 10- to 12-week-old male and female C57BL/6J mice were used. All experimental procedures were approved by the Institutional Animal Care and Use Committee and were conducted in full accordance with the National Institutes of Health Guide for the Care and Use of Laboratory Animals.

### Drugs

Ketamine-hydrochloride (Sigma-Aldrich), MRK-016 (Tocris), and fluoxetine hydrochloride (FLX; National Institute of Mental Health Chemical Synthesis and Drug Supply Program) were dissolved in DMSO and administered intraperitoneally in a volume of 1.125 ml/kg of body mass. 2,3-Dioxo-6-nitro-1,2,3,4-tetrahydrobenzo[*f*]quinoxaline-7-sulfonamide (NBQX; National Institute of Mental Health Chemical Synthesis and Drug Supply Program) was dissolved in 0.9% saline and administered intraperitoneally in a volume of 7.5 ml/kg of body mass. Ketamine was administered at 10 mg/kg, a widely used sub-anesthetic dose widely used in rodent studies to induce antidepressant effects (Maeng et al., 2008; Zanos et al., 2016). Unless stated otherwise, MRK-016 was administered at 3 mg/kg, a dose chosen to produce 70% receptor occupancy (Atack et al., 2009). MRK-016 has equivalent affinity for α1-, α2-, α3-, and α5-containing GABA_A_Rs but has a 5- to 10-fold greater efficacy at inhibiting GABA_A_Rs containing α5 subunits (Atack et al., 2009).

### Chronic restraint stress (CRS)

Male C57BL/6J mice placed in perforated restraint tubes, restricting their movement for 4 h daily with the constant background noise of a biological safety cabinet under blinking fluorescent lighting for a total of 10 d. Mice were individually housed throughout the CRS protocol.

### Behavioral tests

All behavioral experiments were performed during the light phase and experimenters were blinded to the treatment groups during the experimental and analysis phases.

#### Forced-swim test (FST)

Vehicle (DMSO), ketamine (10 mg/kg), or MRK-016 (3 mg/kg) was injected, and mice were tested in the FST at 1 and 24 h postinjection. During the FST, mice were subjected to a 6-min swim session in clear Plexiglas cylinders (30-cm height × 20-cm diameter) filled with 15 cm of water (23 ± 1°C). The FST was performed in normal light conditions (∼800 lux). Sessions were recorded using a digital video camera. Immobility time, defined as passive floating with no additional activity other than that necessary to keep the animal’s head above water, was scored for the last 4 min of the 6-min test by a trained observer blind to the treatment groups. The involvement of AMPARs was determined with the injection of the selective antagonist NBQX (10 mg/kg), administered 10 min before injection of vehicle (DMSO) or MRK-016 (3 mg/kg).

#### Female urine sniffing test (FUST)

Before the start of CRS regimen, mice were tested in the FUST as a measure of a baseline anticipatory hedonic behavior (Malkesman et al., 2010; Zanos et al., 2016). Male C57BL/6J mice were singly-housed in freshly-made home cages for a habituation period of 10 min. Subsequently, one plain cotton tip was secured on the center of the cage wall and mice habituated to the tip for a period of 30 min. Then, the plain cotton tip was removed and replaced by two cotton tip applicators, one infused with fresh female mouse estrous urine and the other with fresh male mouse urine. These applicators were presented and secured at the two corners of the cage wall simultaneously. Sniffing time for both female and male urine was scored by a trained observer for a period of 3 min. Twenty-four hours following completion of CRS, mice were retested in the FUST to assess for the development of an anhedonic phenotype (<70% female urine preference). A total of 37 out of 40 mice were anhedonic by this criteria. Twenty-four hours later, anhedonic mice received an injection of vehicle (DMSO), FLX (20 mg/kg), ketamine (10 mg/kg), or MRK-016 (3 mg/kg) and were retested in the FUST 48 h later.

#### Open-field test

Mice were placed into individual open-field arenas (50-cm length × 50-cm width × 38 cm-height; San Diego Instruments) for a 60-min habituation period. Following the habituation period, vehicle (DMSO), ketamine (10 mg/kg), or MRK-016 (3 mg/kg or 9 mg/kg) was administered, and mice were immediately placed back into the open-field arenas for another 60 min. Sessions were recorded through overhead digital video cameras. Distance traveled was analyzed using TopScan v2.0 (CleverSys).

#### Conditioned-place preference (CPP)

The CPP apparatus consisted of a rectangular three-chambered box (40-cm length × 30-cm width × 35-cm height; Stoelting) comprised of two equal sized end-chambers (20 × 18 × 35 cm) and a central chamber (20 × 10 × 35 cm). One end-chamber had a perforated floor, whereas the other end-chamber had a smooth floor. The CPP protocol consisted of a preconditioning phase, eight conditioning sessions and a postconditioning test. On d 1 (preconditioning phase), mice were placed in the CPP apparatus and were allowed to explore all compartments for a period of 20 min. During the morning sessions of the conditioning phase (d 2-5), vehicle (DMSO) was administered, and mice were placed in their preferred compartment (defined during the preconditioning phase) for 30 min. Five hours later (afternoon sessions), vehicle (DMSO), ketamine (10 mg/kg), or MRK-016 (3 mg/kg) was administered, and mice were placed into their less preferred compartment for 30 min. During the postconditioning test session (i.e., d 6), mice were placed in the CPP apparatus to freely explore all three compartments for 20 min. Time spent in each compartment was measured during the last 15 min of both pre- and postconditioning sessions, as done elsewhere (e.g., Zanos et al., 2014).

#### Prepulse inhibition (PPI)

Mice were individually tested in acoustic startle boxes (SR-LAB, San Diego Instruments). The animals first received an injection of vehicle (DMSO), ketamine (30 mg/kg), or MRK-016 (3 or 9 mg/kg) and were placed in the startle chambers for a 25-min habituation period. The dose of ketamine used for this experiment (30 mg/kg), which is the minimal sub-anesthetic effective dose that results in PPI deficits in CD-1 mice (Zanos et al., 2015). Following a further 5-min adaptation period during which the mice were exposed to a constant background noise (67 dB), five initial startle stimuli (120 dB, 40-ms duration each) were presented. Subsequently, animals were exposed to five different trial types: pulse-alone trials (120 dB, 40-ms duration), three prepulse trials of 76, 81 and 86 dB of white noise bursts (20-ms duration) preceding a 120 dB pulse by 100 ms, and background (67 dB) no-stimuli trials. Each of these trials was randomly presented five times. The percentage PPI (% PPI) was calculated using the following formula: [(magnitude on pulse alone trial – magnitude on prepulse + pulse trial)/magnitude on pulse alone trial] × 100.

#### Rota-rod

The rota-rod test was conducted to compare the effects of ketamine and MRK-016 on motor coordination. The experiment consisted of two phases over a 5-d period: training phase (d 1-4) and a test phase (d 5). On each of the training days, five trials (trial time: 180 s) were conducted with an inter-trial interval of 120 s. Mice were individually placed on the rota-rod apparatus (IITC Life Science) and the rotor (3.75-inch diameter) accelerated from 5-20 RPM over a period of 180 s. Latency to fall was recorded for each trial. Animals with an average of <100 s of latency to fall during the last training day (d 4) were excluded from the experiment and were not treated (*n* = 3 for the saline (SAL) vs DMSO experiment and *n* = 2 for the MRK-016 experiment were excluded). On the test day (d 5), mice received injections of vehicle (DMSO), ketamine (10 mg/kg), or MRK-016 (3 or 9 mg/kg) and were tested on the rota-rod at 5-, 10-, 15-, 20-, 30- and 60-min postinjection using the same process described for the training days.

### Electroencephalogram (EEG)

#### Surgery

Mice were anesthetized with isoflurane (3.5%) and maintained under anesthesia (2-2.5%) throughout the surgery. Mice received analgesia (carprofen, 5 mg/kg, i.p.) before the start of surgery. A PhysioTel miniature implantable radio-telemetric transmitter (Data Sciences International) was inserted subcutaneously and its leads implanted over the dura above the frontal cortex (1.7 mm anterior to bregma) and the cerebellum (6.4 mm posterior to bregma). Animals recovered from surgery for 7 d before recordings.

#### EEG recordings

For assessing the effects of MRK-016 on EEG oscillations, mice were singly-housed and acclimated to the behavioral room for 24 h before EEG recordings. EEGs were recorded using the Dataquest A.R.T. acquisition system (Data Sciences International) with frontal EEG recordings referenced to the cerebellum. Baseline EEG (30 min) recordings were followed by an intraperitoneal injection of saline or NBQX (10 mg/kg), followed 20 min later by an injection of MRK-016 (3 mg/kg).

#### EEG analysis

EEGs were analyzed using Neuroscore (Data Sciences International; Papazoglou et al., 2016). Power in each bandwidth (δ = 1–3 Hz; θ = 4–7 Hz; α = 8–12 Hz; β = 13–29 Hz; γ = 30–80 Hz) was computed in 10-min bins for each animal, normalized to its own mean baseline power.

### Statistical analyses

All statistical analyses were performed using GraphPad v6 (GraphPad Software). For the analysis of the MRK-016 1-hour FST, one-way ANOVA was performed. FUST and CPP data were analyzed using two-way repeated measures ANOVA for factors “treatment” and “experimental phase” (repeated factor). EEG oscillations, open-field test, and rota-rod analyses were performed using two-way repeated measures ANOVA for factors treatment and “time” (repeated factor). For the FST testing NBQX interactions with MRK-016, we used a two-way ANOVA analysis with factors “pretreatment” and treatment. The PPI results were analyzed using two-way repeated measures ANOVA with factors treatment and “dB.” Startle amplitude results were analyzed by one-way ANOVA. All statistical tests were two-tailed, and significance was assigned at *p* < 0.05. ANOVAs were followed by a Holm-Šídák *post hoc* when significance was reached. Main statistical results are presented in Table 1, while *post hoc* comparisons are detailed in Results section and figures.

**Table 1:** Statistical analyses

		Graph	Statistical test	Power	Factor effect				Interaction effect	
		Antidepressant effects			Factor treatment		Factor experimental phase			
**a.**	Fig. 1A	FST	One-way ANOVA	*n* = 10	*F*_(2,27)_ = 16.20;	*p* < 0.001				
**b.**	Fig. 1C	FUST controls	Two-way RM ANOVA	*n* = 8	*F*_(1,14)_ = 0.11;	*p* > 0.05	*F*_(2,28)_ = 3.09;	*p* > 0.05	*F*_(2,28)_ = 0.03;	*p* > 0.05
**c.**	Fig. 1B	FUST	Two-way RM ANOVA	*n* = 9-10	*F*_(3,33)_ = 2.91;	*p* < 0.05	*F*_(2,66)_ = 39.97;	*p* < 0.001	*F*_(6,66)_ = 2.34;	*p* = 0.05
		Effects on cortical oscillations			Factor pretreatment		Factor time			
**d.**	Fig. 2C	γ power	Two-way RM ANOVA	*n* = 8-9	*F*_(1,15)_ = 20.07;	*p* < 0.001	*F*_(11,165)_ = 8.49;	*p* < 0.001	*F*_(11,165)_ = 7.05;	*p* < 0.001
**e.**	Fig. 2D	α power	Two-way RM ANOVA	*n* = 8-9	*F*_(1,15)_ = 2.71;	*p* > 0.05	*F*_(11,165)_ = 2.09;	*p* < 0.05	*F*_(11,165)_ = 2.21;	*p* < 0.05
**f.**	Fig. 2E	β power	Two-way RM ANOVA	*n* = 8-9	*F*_(1,15)_ = 2.83;	*p* > 0.05	*F*_(11,165)_ = 2.12;	*p* < 0.05	*F*_(11,165)_ = 1.29;	*p* > 0.05
**g.**	Fig. 2F	δ power	Two-way RM ANOVA	*n* = 8-9	*F*_(1,15)_ = 0.0001;	*p* > 0.05	*F*_(11,165)_ = 0.64;	*p* > 0.05	*F*_(11,165)_ = 0.81;	*p* > 0.05
**h.**	Fig. 2G	θ power	Two-way RM ANOVA	*n* = 8-9	*F*_(1,15)_ = 2.24;	*p* > 0.05	*F*_(11,165)_ = 2.09;	*p* < 0.05	*F*_(11,165)_ = 1.43;	*p* > 0.05
		NBQX effects, FST			Factor treatment		Factor pretreatment			
**i.**	Fig. 3	1 h postinjection	Two-way ANOVA	*n* = 9	*F*_(1,24)_ = 10.75;	*p* < 0.01	*F*_(1,24)_ = 3.92;	*p* = 0.06	*F*_(1,24)_ = 8.87;	*p* < 0.01
**j.**		24 h postinjection	Two-way ANOVA	*n* = 9	*F*_(1,24)_ = 8.82;	*p* < 0.01	*F*_(1,24)_ = 2.37;	*p* > 0.05	*F*_(1,24)_ = 6.95;	*p* < 0.05
		Effects DMSO on behavior			Factor treatment		Factor time			
**k.**	Fig. 4A	Open-field test (timeline)	Two-way RM ANOVA	*n* = 9	*F*_(1,16)_ = 0.37;	*p* > 0.05	*F*_(23,368)_ = 53.80;	*p* < 0.001	*F*_(23,368)_ = 1.20;	*p* < 0.01
**l.**		Open-field test (bar graph)	Unpaired *t* test (two-tailed)	*n* = 9	*t* = 0.15; df = 16	*p* > 0.05				
**m.**	Fig. 4B	FST	Unpaired *t* test (two-tailed)	*n* = 10	*t* = 0.45; df = 18	*p* > 0.05				
					Factor treatment		Factor dB			
**n.**	Fig. 4C	PPI	Two-way RM ANOVA	*n* = 17	*F*_(1,32)_ = 0.001	*p* > 0.05	*F*_(2,64)_ = 16.18;	*p* < 0.001	*F*_(2,64)_ = 0.80;	*p* > 0.05
**o.**	Fig. 4D	Startle amplitude	Unpaired *t* test (two-tailed)	*n* = 17	*t* = 1.0; df = 28	*p* > 0.05				
					Factor treatment		Factor time			
**p.**	Fig. 4E	Rota-rod	Two-way RM ANOVA	*n* = 8	*F*_(1,15)_ = 0.30;	*p* > 0.05	*F*_(5,75)_ = 1.36;	*p* < 0.001	*F*_(5,75)_ = 1.15;	*p* < 0.01
		Side effects			Factor treatment		Factor time			
**q.**	Fig. 5A	Open-field test (timeline)	Two-way RM ANOVA	*n* = 8	*F*_(1,24)_ = 8.82;	*p* < 0.01	*F*_(1,24)_ = 2.37;	*p* > 0.05	*F*_(1,24)_ = 6.95;	*p* < 0.05
**r.**		Open-field test (bar graph)	Unpaired *t* test (two-tailed)	*n* = 8	*F*_(1,24)_ = 8.82;	*p* < 0.01	*F*_(1,24)_ = 2.37;	*p* > 0.05	*F*_(1,24)_ = 6.95;	*p* < 0.05
**s.**	Fig. 5B	Rota-rod	Two-way RM ANOVA	*n* = 8-9	*F*_(3,30)_ = 12.28;	*p* < 0.001	*F*_(5,150)_ = 11.46;	*p* < 0.001	*F*_(15,150)_ = 10.78;	*p* < 0.001
**t.**	Fig. 5C	CPP	Two-way RM ANOVA	*n* = 10	*F*_(2,27)_ = 3.32;	*p* = 0.05	*F*_(1,27)_ = 0.80;	*p* > 0.05	*F*_(2,27)_ = 7.33;	*p* < 0.01
					Factor treatment		Factor dB			
**u.**	Fig. 5D	PPI	Two-way RM ANOVA	*n* = 6-8	*F*_(3,24)_ = 7.42;	*p* < 0.01	*F*_(2,48)_ = 19.47;	*p* < 0.001	*F*_(6,48)_ = 0.62;	*p* > 0.05

## Results

### Antidepressant effects of MRK-016 in the FST

**Figure 1.**
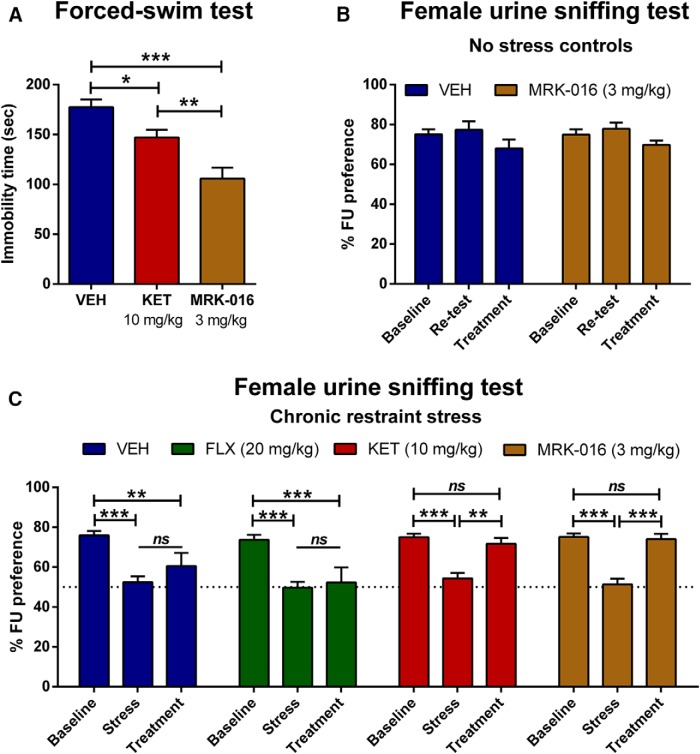
Antidepressant effects of MRK-016 in the forced-swim and FUST. ***A***, Mice received intraperitoneal injections of vehicle (VEH), ketamine (KET) or MRK-016 and were tested in the FST 1 h posttreatment. Acute administration of KET (*n* = 10) and MRK-016 (*n* = 10) significantly reduced immobility in the FST compared with VEH-treated (*n* = 10) mice; one-way ANOVA followed by Holm-Šídák multiple comparison. ***B***, Unstressed control mice displayed no significant change in preference for female urine in two repeated baseline tests or after acute administration of vehicle or MRK-801. ***C***, To investigate MRK-016’s antianhedonic properties, male mice that lost their preference for sniffing female urine following a 10-d restraint stress protocol received VEH (*n* = 10), fluoxetine (FLX; *n* = 9), KET (*n* = 9), or MRK-016 (*n* = 9) and were retested in the FUST 48 h later. Administration of KET and MRK-016 reversed the anhedonia phenotype; two-way repeated measures ANOVA followed by Holm-Šídák *post hoc* test. Data are the mean ± SEM. *p < 0.05, **p < 0.01, ***p < 0.001.

To assess properties of MRK-016 in a classical test of antidepressant efficacy, mice were tested in the FST 1 h postinjection, using ketamine as a positive control (Browne and Lucki, 2013). Both ketamine (10 mg/kg; *p* < 0.05) and MRK-016 (3 mg/kg; *p* < 0.001) administration significantly decreased immobility time in the FST, as compared with the vehicle-treated controls (Fig. 1*A*).

### Rapid antianhedonic properties of MRK-016 in the FUST

To assess the antianhedonic effects of MRK-016 in an anticipatory behavioral test, mice were tested for their preference for female urine after 10 d of restraint stress. MRK-016 or vehicle injection had no effect on preference for female urine in unstressed control mice (Fig. 1*B*), but a single administration of MRK-016 (3 mg/kg) or ketamine (10 mg/kg) 48 h before testing rapidly reversed loss in female urine preference in mice previously subjected to CRS (MRK-016: *p* < 0.001; KET: *p* < 0.01; Fig. 1*C*). Pretreatment with vehicle or a single administration of fluoxetine (20 mg/kg) failed to restore preference for female urine in anhedonic mice (stress vs treatment: *p* > 0.05).

### MRK-016 administration enhances γ power EEG oscillations

**Figure 2.**
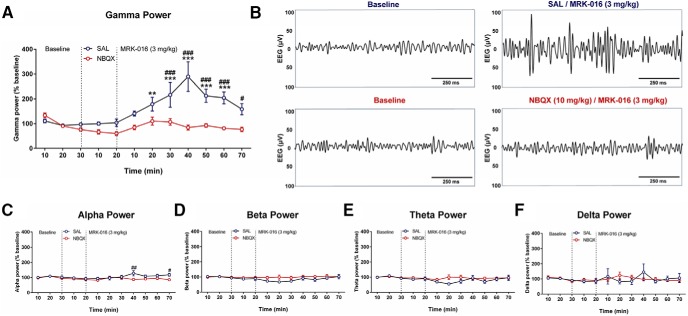
NBQX administration prevents MRK-016 -induced oscillatory EEG activity *in vivo*. Administration of MRK-016 increased γ (***A***, ***B***) and α (***C***) oscillations *in vivo* after prior injection of saline but not after prior injection of NBQX. MRK-016 had no significant effect on β, δ, or θ activity under either condition. Two-way repeated measures ANOVA followed by Holm-Šídák *post hoc* test (vehicle: *n* = 8; MRK-016: *n* = 9). Data are the mean ± SEM. *p < 0.05, **p < 0.01, ***p < 0.001 versus baseline; #*p* < 0.05, ##*p* < 0.01 versus vehicle.

The induction of rapid antidepressant actions of ketamine and its active metabolite (2R,6R)-HNK is accompanied by an increase in the power of EEG activity, particularly at γ frequencies (Hunt et al., 2011; Zanos et al., 2016). Furthermore, the increase in γ activity induced by (2R,6R)-HNK can be prevented selectively by systemic administration of the AMPAR antagonist NBQX (Zanos et al., 2016). We therefore asked whether MRK-016 would induce a similar AMPAR-dependent increase in γ power by administering either NBQX (10 mg/kg) or saline vehicle 20 min before injection of MRK-016 (3 mg/kg). As apparent from the EEG spectrograms from electrodes positioned over the frontal cortex, MRK-016 injection produced a large, persistent increase in EEG power in the γ frequency band over the 10-50 min period after injection (*p* < 0.01; Fig. 2*A*,*B*), without affecting power in other bands [treatment effect: α (*p* < 0.05; Fig 2*C*), β (*p* > 0.05; Fig. 2*D*), θ (*p* > 0.05; Fig. 2*E*), or δ (*p* > 0.05; Fig. 2*F*)]. As for (2R,6R)-HNK (Zanos et al., 2016), MRK-016 induced no significant increase in EEG activity in any frequency band when AMPARs were inhibited by prior injection of NBQX (Fig. 2). Ketamine, its metabolites, and MRK-016 thus induce a common AMPAR-dependent activation of cortical EEG activity, particularly in the γ frequency band.

### An AMPAR antagonist prevents the acute and sustained antidepressant effects of MRK-016

**Figure 3.**
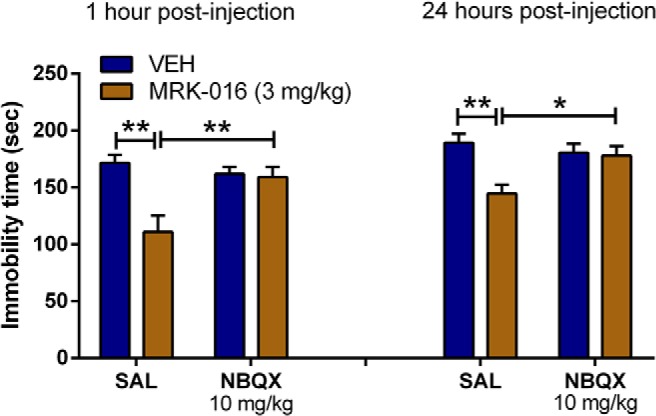
Preadministration of the AMPAR antagonist NBQX prevented the antidepressant effects of MRK-016. NBQX pretreatment 10 min before drug injection prevented the antidepressant-like effects of MRK-016 in the FST as determined 1 h postinjection and 24 h postinjection; two-way ANOVA followed by Holm-Šídák *post hoc* test (SAL/VEH: *n* = 7; SAL/MRK-016: *n* = 7; NBQX/VEH: *n* = 7; NBQX/MRK-016: *n* = 7). Data are the mean ± SEM. *p < 0.05, **p < 0.01.

The induction of persistent antidepressant actions of ketamine and its active metabolites can be prevented by inhibiting AMPARs at the time ketamine is administered (Zanos et al., 2016). To assess mechanistically whether the antidepressant properties of MRK-016 also require AMPARs, we administered the antagonist NBQX (10 mg/kg) 10 min before treatment with vehicle (VEH) or MRK-016 and then assessed the immobility time in the FST 60 min and 24 h later. As reported previously for ketamine, pretreatment with NBQX prevented both the acute (1-h) antidepressant effects of MRK-016 (SAL/MRK-016 vs SAL/VEH: *p* < 0.01; SAL/MRK-016 vs NBQX/MRK-016: *p* < 0.01; Fig. 3), as well as sustained antidepressant actions of the compound observed 24 h after injection (SAL/MRK-016 vs SAL/VEH: *p* < 0.01; SAL/MRK-016 vs NBQX/MRK-016: *p* < 0.05; Fig. 3).

These data indicate that the α5 subunit-selective α5 GABA-NAM MRK-016 shares many of the rapid antidepressant properties of ketamine. Ketamine’s utility as an antidepressant may be compromised by its well-known psychomotor activating, psychotomimetic, and rewarding side effects (Morgan et al. 2012). We therefore asked whether MRK-016 would also produce comparable actions to ketamine in a variety of well-established behavioral assays of these side effects.

### Effects of DMSO on behavior

**Figure 4.**
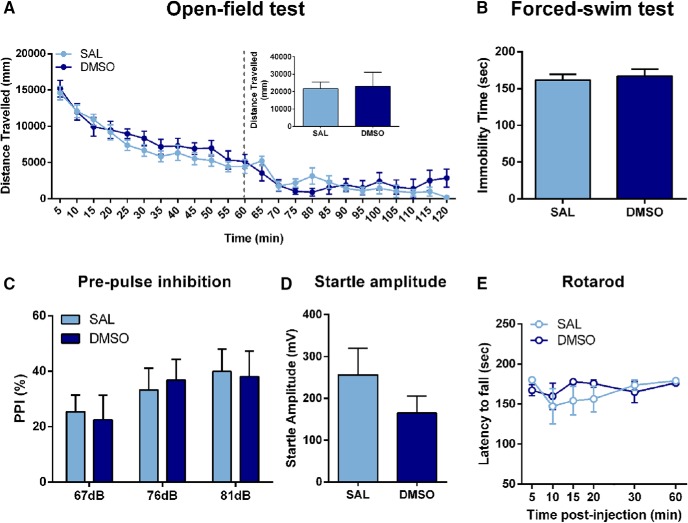
Administration of DMSO does not alter baseline behavioral outcomes in mice. Administration of DMSO did not alter (***A***) locomotor activity (*n* = 9/group), (***B***) immobility time in the FST (*n* = 10/group), (***C***) PPI (*n* = 17/group), (***D***) startle amplitude (*n* = 17/group), or (***E***) motor coordination (SAL: *n* = 8; DMSO: *n* = 9) compared with saline-treated mice. Data are the mean ± SEM.

Because MRK-016 requires DMSO as a solvent, we first tested whether DMSO altered behaviors of mice at the volume used, compared with saline. DMSO administration at 1.125 ml/kg did not alter locomotor activity of mice (*p* > 0.05; Fig. 4*A*), immobility time in the FST (*p* > 0.05; Fig. 4*B*), PPI (*p* > 0.05; Fig. 4*C*), startle amplitude (*p* > 0.05; Fig. 4*D*), or motor coordination (*p* > 0.05; Fig. 4*E*) compared with the saline controls.

### MRK-016 does not induce psychomotor activation

**Figure 5.**
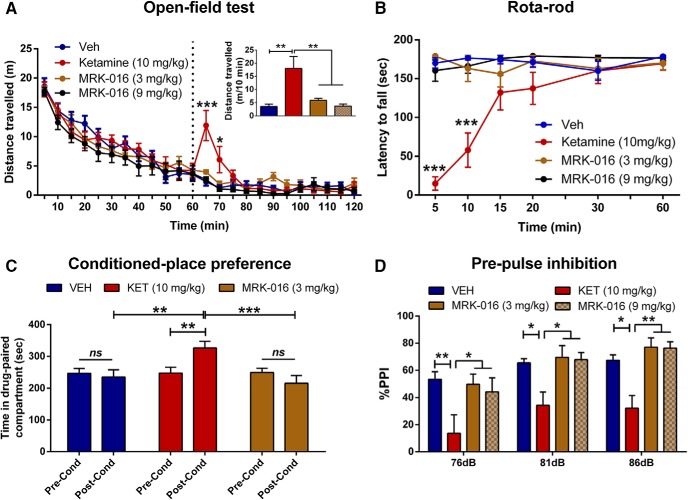
Administration of MRK-016 does not elicit ketamine-like side effects. ***A***, After recording baseline activity for 1 h, mice received drug (marked by a vertical dashed line), and locomotor activity was monitored for another 1 h. Administration of ketamine increased locomotor activity, while administration of vehicle or MRK-016 at two doses did not. ***B***, Unlike ketamine, MRK-016 did not disrupt motor coordination of mice in the rota-rod test; two-way repeated measures ANOVA followed by Holm-Šídák *post hoc* test; (*n* = 9/treatment group). ***C***, Ketamine treatment induced a place preference, revealed as an increased time spent in the drug-paired compartment during the postconditioning (Post-Cond) phase compared with preconditioning (Pre-Cond). However, MRK-016 did not elicit any conditioning in the CPP paradigm; two-way repeated measures ANOVA followed by Holm-Šídák *post hoc* test; (*n* = 10/treatment group). ***D***, Administration of ketamine (*n* = 7; 30 mg/kg) was associated with a disruption of %PPI, whereas MRK-016 at 3 mg/kg (*n* = 7) or 9 mg/kg (*n* = 8) did not affect %PPI compared with control (*n* = 6); two-way repeated measures ANOVA followed by Holm-Šídák *post hoc* test. Data are the mean ± SEM. *p < 0.05, **p < 0.01, ***p < 0.001.

Many psychostimulants, like ketamine, produce hyperlocomotion in an open field (Irifune et al., 1991). Indeed, acute injection of ketamine (10 mg/kg) significantly increased locomotor activity within minutes, whereas administration of MRK-016 (3 or 9 mg/kg) did not (Fig. 5*A*).

### MRK-016 does not disrupt motor coordination

A common side effect of ketamine in humans, and rodents is dizziness and loss of coordination (Ghoneim et al., 1985). Acute administration of MRK-016 at the doses of 3 and 9 mg/kg did not affect the motor coordination of mice in the rota-rod test, compared with the vehicle-treated mice (*p* > 0.05). In contrast, ketamine (10 mg/kg) disrupted motor coordination of mice 5 min (*p* < 0.001 vs all the other treatments) and 10 min after injection (*p* < 0.001 vs all the other treatments; Fig. 5*B*).

### MRK-016 does not elicit CPP

In rodents, administration of drugs with known risk for human abuse, including ketamine (Suzuki et al., 1999), often elicits a behavioral preference for the place where they are administered, i.e., a CPP. Ketamine-treated (10 mg/kg) mice exhibited a robust CPP, as indicated by the increased time spent in the drug-paired compartment during the postconditioning test compared with the preconditioning phase (*p* < 0.01; Fig. 5*C*). In contrast, administration of MRK-016 (3 mg/kg) did not induce CPP (*p* > 0.05; Fig. 5*C*).

### MRK-016 does not disrupt PPI

In rodents and humans, administration of some psychotomimetic drugs inhibits the ability of a weak conditioning tone to suppress the startle response to a loud tone, i.e., PPI. As reported previously (Mansbach and Geyer, 1991), ketamine administration at 30 mg/kg disrupted PPI in response to conditioning tones of 76 dB (*p* < 0.01), 81 dB (*p* < 0.05), and 86 dB (*p* < 0.05), compared with the vehicle-treated control mice (Fig. 5*D*). In contrast, MRK-016 administration did not alter PPI at any prepulse intensity (*p* > 0.05; Fig. 5*D*) compared with controls at both the dose at which it elicits an antidepressant-like effect (3 mg/kg) and at three times that dose (9 mg/kg). Neither ketamine nor MRK-016 altered the startle amplitude of the animals compared with vehicle-treated controls (VEH: 200.8 ± 47.4; KET: 181.5 ± 47.4; MRK-016 3 mg/kg: 314.8 ± 59.0; MRK-016 9 mg/kg: 330.7 ± 73.5; *p* > 0.05).

## Discussion

There is considerable need for new, improved antidepressant drug therapies that produce more rapid relief of depressive symptoms and are more widely effective than SSRIs. Although ketamine has clinical efficacy, its potential for causing negative side effects due to NMDAR inhibition is a significant limitation for its widespread use as a next-generation antidepressant. Nevertheless, ketamine has provided an invaluable tool drug for understanding how rapid relief of depressive symptoms may be produced. Although many mechanistic details remain to be determined, there is general agreement that ketamine ultimately acts to strengthen excitatory synapses in the prefrontal cortex, hippocampus, ventral striatum, and other brain regions critically involved in the regulation of mood and reward (Li et al., 2010; Autry et al., 2011; Zanos et al., 2016); synapses that become weakened in response to prodepressive conditions such as chronic stress (Pittenger and Duman, 2008; Lim et al., 2012; Yuen et al., 2012; Kallarackal et al., 2013; Thompson et al., 2015).

Negative allosteric modulators of GABA_A_Rs containing α5 subunits, such as MRK-016, which act as a partial inverse agonists at the benzodiazepine binding site and are highly selective for receptors containing α5 subunits, reverse the behavioral signs of consumatory anhedonia, as assayed with the sucrose preference test, produced by multiple chronic stress paradigms within 24 h of a single systemic administration and that the beneficial antianhedonic effects persist for several days (Fischell et al., 2015). We extend these previous results here by showing that α5 GABA-NAMs also exert an antidepressant-like response in the forced swim test, both acutely and 24 h later. We also demonstrate that their antianhedonic actions extend into anticipation of reward, as assayed by the preference of males for the scent of urine from female mice. The restoration of normal behavior by α5 GABA-NAMs in chronic stress models is likely mediated by a restoration of excitatory synaptic strength at stress-sensitive synapses in the cortico-mesolimbic circuits (Fischell et al., 2015).

It has been suggested that ketamine and/or its hydroxynorketamine metabolites restore synaptic strength and relieve the symptoms of depression by promoting increases in coherent neuronal discharge in the neocortex and hippocampus, either indirectly through disinhibition (Farber et al., 1998; Homayoun and Moghaddam, 2007; Dwyer and Duman, 2013) or directly by potentiating excitatory synapses (Zanos et al., 2016). Indeed, the persistent antidepressant-like actions of ketamine and its metabolite (*2R,6R*)-hydroxynorketamine are accompanied by a transient period of EEG activation, particularly at γ frequencies (30-60 Hz), while the compounds are present in the brain. If this transient EEG activation is prevented by simultaneous inhibition of AMPARs with NBQX, then ketamine and its metabolite fail to induce any antidepressant-like behavioral effects (Maeng et al., 2008; Autry et al., 2011; Zanos et al., 2016). Similar to ketamine and its active metabolites, MRK-016 induced a significant increase in coherent EEG at γ frequencies and both the EEG activation and persistent antidepressant-like response to MRK-016 in the FST were prevented by coadministration of NBQX. Although ketamine and α5 GABA-NAMs act at different targets, it appears that the two classes of compounds converge onto a common mechanism of antidepressant action. We suggest that the coherent γ frequency oscillations produced by both classes of compounds are accompanied by coherent pre- and postsynaptic neuronal activity in the cortex and hippocampus that is known to promote activity-dependent synaptic potentiation, hence accounting for the persistence of the behavioral response long after the compounds have been eliminated from the brain.

Ketamine inhibits NMDARs that are widespread throughout the brain. This lack of target localization is likely to contribute to its psychomotor activating, abuse potential, cognition disrupting, and psychomimetic properties. Because GABA_A_R α5 subunits display a relatively discrete expression within the cortex and hippocampus (Malherbe et al., 1990; Persohn et al., 1992), we predicted that they would exert fewer side effects than ketamine. Indeed, we observed no effects of MRK-016 on ketamine-sensitive behaviors such as locomotion in an open field, motor coordination on the rota-rod, or the ability of a prepulse tone to inhibit the startle response. In addition, MRK-016 administration failed to induce a place preference, indicating a low abuse potential, unlike ketamine. MRK-016 has previously been administered to healthy human volunteers and did not induce significant side effects, such as dizziness or anxiety, except at high doses (Atack et al., 2009). Finally, unlike the cognitive defects associated with chronic ketamine use (e.g., Morgan et al. 2012), α5 GABA-NAMs promote learning and memory in animal models (Ballard et al., 2009) and are under development as cognitive enhancers (Rudolph and Knoflach, 2011).

Alpha**-**5 subunit-selective negative allosteric modulators of GABA_A_Rs thus display a combination of ketamine-like rapid and persistent antidepressant and antianhedonic effects, with a favorable side effect profile, compared with ketamine. We therefore suggest that this class of compounds may represent a novel, fast-acting, effective, and clinically viable treatment for human depression.

## Synthesis

Reviewing Editor: Quentin Pittman, University of Calgary Decisions are customarily a result of the Reviewing Editor and the peer reviewers coming together and discussing their recommendations until a consensus is reached. When revisions are invited, a fact-based synthesis statement explaining their decision and outlining what is needed to prepare a revision will be listed below. The following reviewer(s) agreed to reveal their identity: Uwe Rudolph, Ronald Duman.

Both reviewers are in principal agreement that this study provides interesting information, both confirmation of antidepressant-like effects of GABAA alpha5 negative allosteric modulators already described in a previous report (J Fischell et al., 2015)and novel data on changes in EEG gamma power.

However, both reviewers have made suggestions for improvement of your manuscript, and after consultation we have all agreed that it will be important that you address all of their concerns. In addition , it is likely that some additional experiments may be required.

With respect to additional experiments:

1 You demonstrate that blockade of AMPA receptors blocks the effects of MRK-016 on immobility in the FST, but do not examine FUST or γ frequency. The manuscript would be improved with addition of these data.

2. Important controls are not included in the FUST study, namely, the influence of drug treatment alone in naïve, non stressed mice.

There are several suggestions for improvement in the text/figures that can be addressed easily in revision:

1) The title is quite unspecific with respect to the mechanism of MRK-016 and thus partially misleading. More, actually throughout the manuscript, the authors should change the term "antidepressant action" to "antidepressant like action. Suggestion for the title: "A negative allosteric modulator selective for 5-containing GABAA receptors, MRK-016, exerts a rapid and persistant antidepressant-like action without the side effects of the NMDA receptor antagonist keatmine in mice."

2) The term "GABA-NAM", which occurs throughout the manuscript, should not be used, as it is highly misleading at least for pharmacologists, although it is correctly introduced and explained in the abstact. There are GABAA receptors and GABAB receptors. It should be clear which of these receptors are modulated. Moreover, it has to be clear which GABAA receptor subtype is selectively modulated. Suggestion: "GABAA alpha5-selective negative allosteric modulator" ("R" for receptor) or "GABAA alpha5-NAM" (Greek alpha symbol), or "alpha5 GABAA-NAM". The short v version "alpha5-NAM" would also work if it is spelled out that this refers to GABAA receoptors.

3) I recommend that the authors provide published information on the selectivity of the compound MRK-016 for alpha5-containing GABAA receptors over other GABAA receptor subtypes anywhere in the manuscript. Is it binding-selective or efficacy-selective? What is the selectivity over other GABAA receptor subtypes ? In other words, binding data and efficacy data from the literature should be included. The conclusion that what the authors are observing is really due to alpha5-containing GABAA receptors rests on the selectivity of this compound for different GABAA receptor subtypes, as defined by their alpha subunits. This information should be provided to the readers of this paper.

4) Page 16, line 7. "Negative allosteric modulators of GABAARs, such as MRK-016" is too imprecise and should thus be avoided. Suggestion: "Negative allosteric modulators of alpha5-containing GABAARs". These receptors can also be called "alpha5-GABAARs" if it is stated once that this abbreviation means alpha5-containing GABAAA receptors, as these receptors contain beta and gamma subunits in addition to alpha5.

5) The authors should make it more clear in the introduction that there have been previous publications with MRK-016, as well as another α5 NAM.

6) Page 4: "negative side effects". Are there also "positive side effects" ? Perhaps replace by "unwanted effects" or "undesired effects".

7) Page 12, second paragraph. The designation of the parts of Figure 2 are incorrect. 2D is really 2C, 2E is really 2D, 2G is really 2E, and 2H is really 2G.

8) Page 16, line 1. Typographical error. Replace "act" with "acts".

9) Fig. 2. The "lines" in the electrogram are hardly visible on the high-quality printout that I made.

10) Page 18, last paragraph of discussion. The last sentence is perhaps going a little too far considering the data. Consider changing "represents" to "may represent".
